# Gene Expression in Spontaneous Experimental Autoimmune Encephalomyelitis Is Linked to Human Multiple Sclerosis Risk Genes

**DOI:** 10.3389/fimmu.2020.02165

**Published:** 2020-09-18

**Authors:** Hans Faber, Dunja Kurtoic, Gurumoorthy Krishnamoorthy, Peter Weber, Benno Pütz, Bertram Müller-Myhsok, Frank Weber, Till F. M. Andlauer

**Affiliations:** ^1^Max Planck Institute of Psychiatry, Munich, Germany; ^2^Max Planck Institute of Biochemistry, Martinsried, Germany; ^3^Institute of Translational Medicine, University of Liverpool, Liverpool, United Kingdom; ^4^Department of Neurology, Klinikum rechts der Isar, School of Medicine, Technical University of Munich, Munich, Germany

**Keywords:** experimental autoimmune encephalomyelitis (EAE), myelin oligodendrocyte glycoprotein (MOG), T helper cell (Th), multiple sclerosis, risk genes, gene expression

## Abstract

Recent genome-wide association studies have identified over 230 genetic risk loci for multiple sclerosis. Current experimental autoimmune encephalomyelitis (EAE) models requiring active induction of disease may not be optimally suited for the characterization of the function of these genes. We have thus used gene expression profiling to study whether spontaneous opticospinal EAE (OSE) or MOG-induced EAE mirrors the genetic contribution to the pathogenesis of multiple sclerosis more faithfully. To this end, we compared gene expression in OSE and MOG EAE models and analyzed the relationship of both models to human multiple sclerosis risk genes and T helper cell biology. We observed stronger gene expression changes and an involvement of more pathways of the adaptive immune system in OSE than MOG EAE. Furthermore, we demonstrated a more extensive enrichment of human MS risk genes among transcripts differentially expressed in OSE than was the case for MOG EAE. Transcripts differentially expressed only in diseased OSE mice but not in MOG EAE were significantly enriched for T helper cell-specific transcripts. These transcripts are part of immune-regulatory pathways. The activation of the adaptive immune system and the enrichment of both human multiple sclerosis risk genes and T helper cell-specific transcripts were also observed in OSE mice showing only mild disease signs. These expression changes may, therefore, be indicative of processes at disease onset. In summary, more human multiple sclerosis risk genes were differentially expressed in OSE than was observed for MOG EAE, especially in T_H_1 cells. When studying the functional role of multiple sclerosis risk genes and pathways during disease onset and their interactions with the environment, spontaneous OSE may thus show advantages over MOG-induced EAE.

## Introduction

Although animal models are widely used in human research, it is still discussed whether they can adequately mirror diseases like multiple sclerosis (MS) that only exist in humans. MS is a chronic inflammatory disease of the central nervous system (CNS), with both environmental and genetic risk factors contributing to disease susceptibility. The recent identification of more than 230 genetic risk loci for MS (1, 2) requires a reassessment of the widely used experimental autoimmune encephalomyelitis (EAE) animal models. To support analyses of the primary cause and etiology of MS, animal models should ideally replicate mechanisms taking place during MS disease induction.

Most EAE models are actively induced by injection of myelin-derived antigens in conjunction with potent adjuvants (3). One such antigen is myelin oligodendrocyte glycoprotein (MOG), a component of the outer surface of myelin (4). Injection of the MOG_35−55_ peptide into *C57BL/6* mice leads to chronic EAE (5) and thus serves as a popular animal model to date. A related model, passively-transferred EAE, is caused by bulk transfer of *in vitro*-activated myelin-specific T cells (6).

By contrast, transgenic models such as opticospinal EAE (OSE) spontaneously develop autoimmune disease and may, therefore, be better suited to study disease onset than induced EAE is. Spontaneous models can be used for identifying environmental triggers of MS (7, 8) and might support analyses of genetic risk factors for human MS. They circumvent problems specific to induced ones, such as adjuvant inoculation, with its partially unknown effects. In OSE, ~50% of the animals develop a spontaneous inflammatory demyelinating CNS disease, predominantly affecting optic nerves and the lumbar part of the spinal cord (9). These mice carry two transgenic modifications: they express a T cell receptor (TCR) recognizing the MOG_35−55_ peptide and B cells with MOG-specific receptors. In OSE, MOG-specific B cells function as antigen-presenting cells to trigger disease onset by activating MOG-specific T cells (10). Notably, B cell-depleting treatments for MS appear to target primarily cellular and not humoral B cell responses, and, thus, result in a reduced T cell activation (11).

For a long time, T_H_1 cells were considered as the predominant drivers of EAE and MS (4). This hypothesis was challenged by emerging evidence for a substantial role of T_H_17 cells in the disease etiology, including the discovery that the transfer of T_H_17 cells can induce EAE. In fact, both T_H_ cell types can induce EAE, albeit with distinct pathologies (12). In humans, genome-wide association studies (GWAS) have identified many MS risk loci that support a central role of T_H_ cells and T_H_ cell differentiation in the pathophysiology of MS (1, 2, 13).

Despite their valuable contributions to our understanding of MS pathophysiology and drug development, the relationship of EAE to human MS remains controversial (14). All available EAE models are, to some degree, artificial. Therefore, knowledge of whether gene expression changes in diseased mice involve MS risk genes can support the choice of an EAE model for specific research projects. The present study had three aims: First, to characterize gene expression differences in diseased OSE and MOG_35−55_ EAE mice, two widely used EAE models with markedly different forms of induction. Second, to explore which of OSE or MOG-induced EAE resembles human MS more closely. To this end, we examined to which degree genes differentially expressed in spinal cord samples of OSE and MOG EAE showed significant enrichment of human MS risk genes. Third, to analyze expression differences of T_H_ cell-specific transcripts in both EAE models.

## Materials and Methods

### Mice, Animal Handling, and Scoring

All mice used in this study had a *C57BL/6* background and were bred in the animal facilities of the Max Planck Institute of Biochemistry and Neurobiology, Martinsried, Germany. For the OSE model, double-transgenic *2D2* (*TCR*_*MOG*_) × *IgH*_*MOG*_ (OSE) mice were used (9). For MOG EAE, wildtype *C57BL/6* mice were immunized subcutaneously with 200 μg of a MOG peptide consisting of the amino acids 35–55, emulsified in complete Freund's adjuvant supplemented with 5 mg/ml *Mycobacterium tuberculosis* (strain H37Ra, Thermo Fischer Scientific BD Difco), as described previously (9). Pertussis toxin (400 ng, List Biological Laboratories) was injected intraperitoneally on the day of immunization and 48 h later. Control mice (CFA) received the same treatment but without the MOG peptide. For the analysis of EAE models, only female mice were used. For the T_H_ cell analyses, OSE mice of mixed gender were used (15).

Scores for clinical signs of EAE were assessed daily according to the standard 5-point scale (9, 16): 0: healthy animal; 1: animal with a flaccid tail; 2: animal with impaired righting reflex and/or gait; 3: animal with one paralyzed hind leg; 4: animal with both hind legs paralyzed; 5: moribund animal or death of the animal after preceding clinical disease. See Supplementary Figure 1 for the disease course of MOG EAE compared to control mice. Following our ethically approved protocol, the mice were sacrificed when they reached a score of 4. The animal welfare committee of the government of Upper Bavaria (Tierschutzkommission der Regierung von Oberbayern, Munich, Germany) approved the protocol. The animal procedures were in strict accordance with the guidelines set down by the animal welfare committee of the government of Upper Bavaria.

### *In vitro* CD4^+^ T Cell Differentiation

T cells derived from the spleen of a mixed-gender pool of four OSE mice were used to polarize pathogenic effector T_H_1 and T_H_17 cells, as described previously (15). In brief, four separate batches of four mice each were used for this experiment. To generate T_H_1 cells, total erythrocyte-lysed spleen cells from OSE mice were cultured in the presence of a MOG peptide (amino acids 1–125), IL-12, IL-18, and anti-IL-4. After 3 days, IL-2 was added to the culture. To generate T_H_17 cells, total erythrocyte-lysed spleen cells from OSE mice were cultured in the presence of a MOG peptide (amino acids 1–125), TGF-β1, IL-6, IL-23, anti-IL-4, and anti-IFN-γ. After 3 days, IL-23 was added to the culture. In both cases, cells were re-stimulated after 6 days and harvested after 9 and, once more, after 12 days. Naïve T_H_0 cells were harvested on day 0. The success of polarization was evaluated by flow cytometry, ELISA, and quantitative real-time PCR (Supplementary Figure 2 and Supplementary Methods).

### Microarrays

Two separate microarray experiments were performed on the Illumina gene expression profiling platform: The first comprised RNA isolated from total spinal cord preparations of healthy and diseased EAE mice. The second experiment analyzed gene expression profiles of naïve T_H_0 cells and *in vitro* polarized T_H_1 and T_H_17 cells. For the analysis of EAE models, the Sentrix BeadChip Array MouseWG-6 v2 (Illumina, San Diego, USA) was used; for the T_H_ cell microarray, the Sentrix BeadChip Array MouseWG-6 v1.1 (Illumina, San Diego, USA). Four chips (24 samples, four per experimental group) were hybridized in the EAE experiment, three chips (18 samples from four separate experiments: 4 × T_H_0, 7 × T_H_1, 7 × T_H_17) were used for the T_H_ cell analysis. In each experiment, all samples and chips were processed in parallel. RNA processing, array hybridization, and quantification followed the same protocols in both experiments: First, concentration and purity of total RNA were assessed by 260 nm UV absorption and by 260/280 ratios, respectively (Nanophotometer, Implen, Munich, Germany). Second, RNA integrity was evaluated using a chip-based electrophoretic assay (Agilent RNA 6000 Nano Kit used in conjunction with the Agilent 2100 bioanalyzer, Agilent Technologies, Waldbronn, Germany). Mean RNA integrity numbers were 8.4 (SD = 0.5) for the EAE and 9.0 (SD = 0.5) for the T_H_ cell experiment. Third, RNA was amplified and labeled using the Illumina TotalPrep RNA Amplification Kit (Ambion, Houston, TX, USA) and hybridized onto Illumina gene expression arrays following the manufacturer's instructions. Fourth, fluorescence signals were scanned on an Illumina BeadStation and analyzed by in-house software routines. The manufacturer's built-in controls were analyzed, including hybridization controls and sample-dependent parameters. All microarrays fulfilled Illumina's recommendations for quality control (QC).

### Quality Control of Microarrays

Raw probe intensities were exported as summary data using Illumina's GenomeStudio, and further statistical processing was carried out using *R* v3.3.2 (17). For the analysis of EAE models, summary data was loaded using the Bioconductor package *beadarray* (18), and QC was conducted with *lumi* (19) and *vsn* (20). Each probe was transformed and normalized through variance stabilization and normalization. Probes were removed if they showed a detection *p*-value < 0.05 in >10% of the samples or had a “no match” or “bad” probe quality in the *illuminaMousev2.db* package. This procedure left 21,483 transcripts from 24 samples. For the T_H_ cell experiment, summary data was loaded using *limma* (21) and QC was conducted with *limma* and *vsn*. Probes were transformed, normalized, and filtered as described above, based on the *illuminaMousev1p1.db* package. This pipeline left 17,858 transcripts. Technical batch effects were examined by inspecting the association of the first ten principal components of expression levels with expression chip and position on the chip.

### Analysis of Differential Expression

Principal component analysis (PCA) was conducted in *R* using the function *prcomp* without scaling of variables; PCs were scaled for display. K-means clustering was performed using *kmeans* with *k* = 4; the analysis was repeated 100 times and the most stable clustering solution was selected. Differential expression was assessed with *limma*. For the analysis of differential expression across the EAE models, six mouse types were examined (with four mice each): wild-type (WT); healthy OSE controls (OSE_0_); OSE with disease score 1 (OSE_1_); OSE score 4 (OSE_4_); as a MOG EAE control, healthy control mice injected with complete Freund's adjuvant but not with a MOG peptide (CFA); as MOG_35−55_ EAE, *C57BL/6* wildtype mice injected with adjuvant and MOG_35−55_ peptide, rated score 4 (MOG_4_). The design matrix was constructed from the six mouse types. Each expression chip contained one sample per mouse type. The four chips were added to the model as random effects via the *duplicateCorrelation* function. The five contrasts MOG_4_-CFA, CFA-WT, OSE_4_-OSE_0_, OSE_1_-OSE_0_, and OSE_4_-WT were computed on the fitted linear model and moderated *t*-tests were calculated using the *eBayes* function. For the T_H_ cell experiment, the design matrix was constructed from the three cell types (naïve T_H_0, T_H_1, T_H_17), with the four mouse pools treated as random effects. Only T_H_1 and T_H_17 cells harvested on day 9 were analyzed. The two contrasts T_H_1-T_H_0 and T_H_17-T_H_0 were examined.

### Overrepresentation Analyses

Overrepresentation analyses (ORA) were conducted using WebGestalt v2019 (22) in *R*, based on the gene ontology (GO) biological process database. Genes were submitted as unique Entrez IDs, and the reference was *genome protein-coding*. The significance level was determined using a hypergeometric test, followed by calculation of the Benjamini-Hochberg false discovery rate (FDR) (23).

### Enrichment Tests

The enrichment of genes was calculated using a permutation test in *R*. For this test, the unique Entrez IDs of genes were used. First, the amount of unique differentially expressed genes was determined, and the same number of random genes was selected. Second, the number of these random genes overlapping with the test set of genes (e.g., MS susceptibility genes) was determined. These two steps were repeated 100,000 times. Third, to calculate a *p*-value, the number of observations where the overlap between random genes and test genes was equal to or larger than the overlap between differentially expressed genes and test genes was counted and divided by the number of permutations.

For the enrichment analysis with MS susceptibility genes, the 558 genes outside the major histocompatibility complex (MHC) region listed in Supplementary Table 18 of the MS genomic map published in 2019 by the IMSGC were used (2). From this list, *CTB-50L17.10, RP11-345J4.5, JAZF1-AS1, ZEB1-AS1, GATA3-AS1, SSTR5-AS1*, and *RPL34-AS1* were excluded to generate the list of 551 prioritized putative MS susceptibility genes described in the IMSGC publication.

## Results

**Figure 1:**
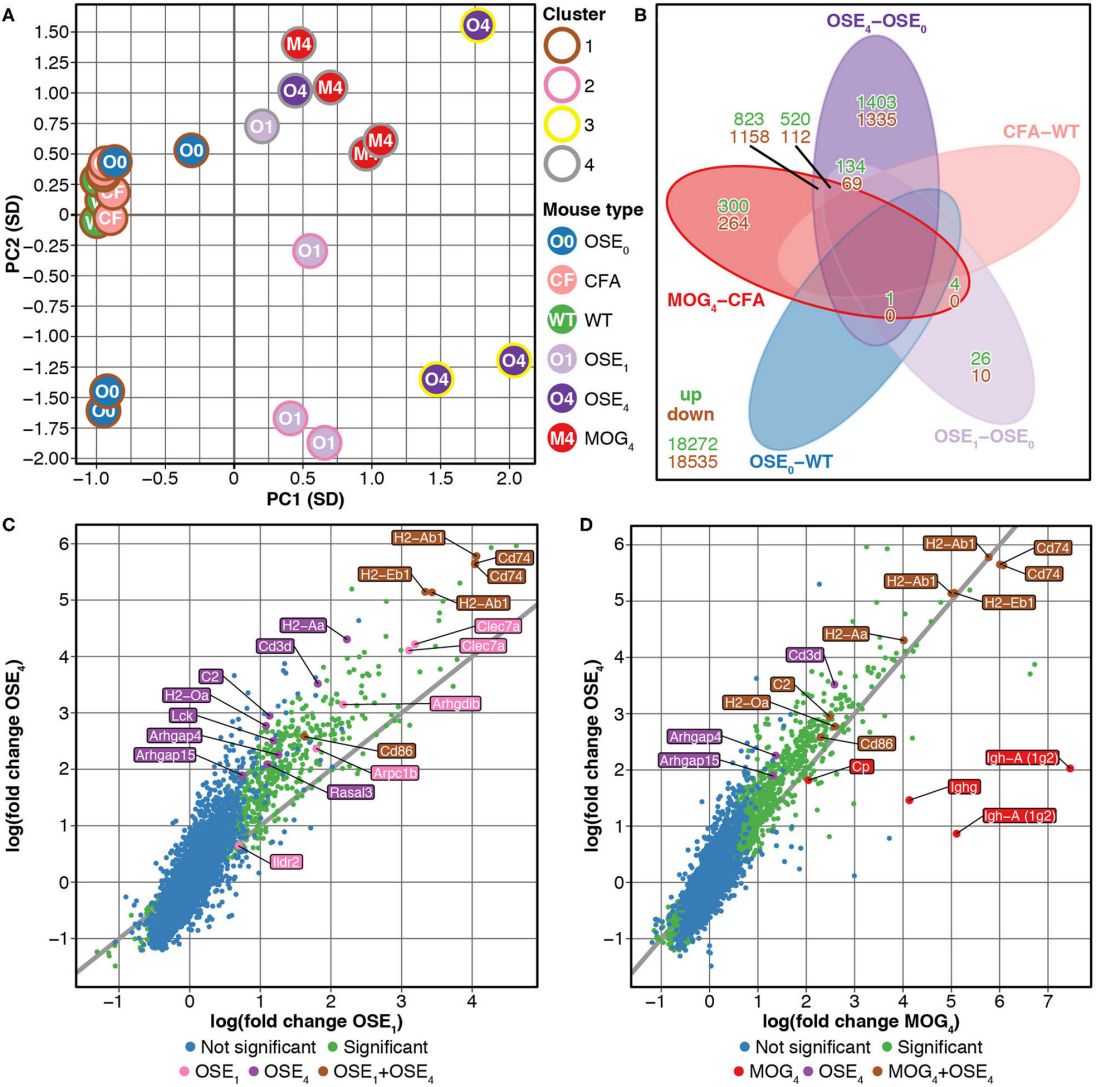
Differential expression analysis of OSE and MOG EAE models. **(A)** Principal component analysis (PCA) of gene expression profiles grouped by k-means clustering. The cluster solution displayed here was the most frequent (34 of 100). In 97 of 100 analyses, all WT, OSE_0_, and CFA mice were placed into separate clusters than OSE_1_, OSE_4_, and MOG_4_ mice (Supplementary Table 1). Because of the spontaneous nature of disease development in OSE mice, gene expression in diseased OSE_4_ animals showed more variance than was, e.g., observed in MOG_4_ mice, which exhibit a more stereotypic disease course (9). PC, principal component; SD, standard deviations. **(B)** Venn diagram highlighting the number of transcripts differentially expressed in the analyzed contrasts. For this plot, up- and downregulated transcripts were analyzed separately, and transcripts differentially expressed in opposing directions are therefore included in the counts. **(C,D)** OSE_4_ mice showed greater fold changes of expression levels than **(C)** OSE_1_ and **(D)** MOG_4_ mice, each compared to its control condition (OSE_0_ and CFA, respectively) (Supplementary Table 3). For each group, the ten most differentially expressed genes (with Entrez IDs) are labeled. If two probes per gene were present among the top differentially expressed transcripts, the gene was counted only once, but both probes are plotted. The groups are: differentially expressed in OSE_1_ only (light magenta), differentially expressed in OSE_4_ only (dark magenta), differentially expressed in MOG_4_ only (red), differentially expressed in **(C)** both OSE_1_ and OSE_4_ or **(D)** both MOG_4_ and OSE_4_ (brown; with higher expression levels observed for OSE_4_).

We compared gene expression profiles of total spinal cord preparations derived from two EAE models, OSE and MOG_35−55_ EAE. Double-transgenic OSE mice developed CNS autoimmunity spontaneously, predominantly affecting the lumbar part of the spinal cord. In the MOG_35−55_ EAE model, the disease was induced in *C57BL/6* wildtype (WT) mice by immunization with a MOG_35−55_ peptide. PCA of gene expression profiles separated healthy (OSE_0_, CFA, and WT) from diseased [OSE score 1 (OSE_1_), OSE score 4 (OSE_4_), and MOG score 4 (MOG_4_)] animals along the first component (Figure 1A). Most variance in gene expression was thus observed between healthy and diseased mice and not between EAE models. Because of the spontaneous nature of disease development in OSE mice, gene expression in diseased OSE_4_ animals showed more variance than was, e.g., observed in MOG_4_ mice, which exhibit a more stereotypic disease course (9).

Unsupervised k-means clustering on PCs further supported this finding, which consistently (in 97 of 100 replications) placed healthy and diseased animals into separate clusters (Figure 1A, Supplementary Table 1). The most frequent cluster solution (34/100) placed all healthy mice together in cluster 1; additional clusters were OSE_1_ only (cluster 2), OSE_4_ only (cluster 3), and a mixed cluster of the remaining diseased animals (cluster 4). We could thus successfully detect disease-relevant gene expression changes in the animals.

### Stronger Gene Expression Changes in the OSE Model

Next, we analyzed gene expression changes in OSE and MOG EAE mice. We examined differential expression for five contrasts: OSE_1_-OSE_0_, OSE_4_-OSE_0_, MOG_4_-CFA, and the two control contrasts CFA-WT and OSE_0_-WT (Figure 1B, Supplementary Table 2). In the control contrast CFA-WT, no transcript was differentially regulated. A single transcript was upregulated in OSE_0_-WT, *T cell receptor alpha chain* (*Tcra*), which was also upregulated in all other contrasts except CFA-WT. The number of significantly up- and downregulated transcripts was higher for OSE_4_-OSE_0_ (*n* = 5,555) than for MOG_4_*-*CFA (*n* = 3,182). In total, the expression of 864 transcripts differed significantly between MOG_4_ and OSE_4_ mice (Supplementary Table 2). Interestingly, 4.88× more transcripts were differentially expressed specifically only in OSE_4_-OSE_0_ than only in MOG_4_*-*CFA (Figure 1B). Moreover, fold changes were higher in OSE_4_-OSE_0_ than in either OSE_1_*-*OSE_0_ (binomial test: *p* = 1.4 × 10^−65^ for all transcripts, *p* = 9.9 × 10^−119^ for transcripts differentially expressed in both contrasts, Figure 1C) or MOG_4_*-*CFA (*p* = 5.8 × 10^−3^ for all, *p* = 2.7 × 10^−221^ for differentially expressed transcripts, Figure 1D; Supplementary Table 3). Stronger global gene expression changes were thus triggered in OSE than in MOG EAE.

### Overrepresentation of Immune System Processes Especially for OSE

To characterize the expression changes in the different EAE models further, we conducted ORA analyses of the analyzed contrasts (Supplementary Table 4, Supplementary Figure 3) and of differentially expressed transcripts for three groups (Supplementary Figure 4): First, *common disease transcripts* (CDT), differentially expressed for both contrasts OSE_4_*-*OSE_0_ and MOG_4_*-*CFA but not in the two control contrasts OSE_0_-WT or CFA-WT. Second, *OSE*_4_*-specific transcripts* (OSE_4_sp), differentially expressed for the contrast OSE_4_*-*OSE_0_ but not for MOG_4_*-*CFA or the control contrasts. Third, *MOG*_4_*-specific transcripts* (MOG_4_sp), differentially expressed for MOG_4_*-*CFA but not for OSE_4_*-*OSE_0_ or the control contrasts. When examining CDT, 1,379 redundant GO biological processes remained significant after correction for multiple testing (Supplementary Table 4). Together with other immune-related gene sets, *immune response, regulation of immune system process*, and *T cell activation* were among the top-associated terms (adjusted *p* < 2 × 10^−16^). These and other immune-associated processes remained significant in OSE_4_sp (adjusted *p* ≤ 3.5 × 10^−2^, Supplementary Figure 4). By contrast, no immune system-specific process was significant for MOG_4_sp. More expression changes in the immune system were, therefore, triggered in OSE than in MOG EAE.

### Activation of the Adaptive Immune System in OSE_1_ Mice

While MOG EAE develops rapidly in a highly stereotypical manner, the clinical course of OSE is usually slower and shows more inter-individual variability (24). OSE thus allows for studying disease at different stages, and we analyzed mice showing a mild disease score of 1 (OSE_1_). Compared to OSE_0_, 34 transcripts were differentially expressed specifically in OSE_1_ animals and not in any other contrast [*OSE*_1_*-specific transcripts* (OSE_1_sp), Supplementary Table 5]. These transcripts are potentially indicative of changes during mild or early disease. However, no significant GO biological processes were identified for them. Transcripts differentially regulated in both OSE_1_ and OSE_4_ consistently showed the same direction of regulation compared to OSE_0_ [binomial test *p* = 4.36 × 10^−252^, 95% confidence interval (CI) 0.995–1.0, Supplementary Table 3]. When analyzing all transcripts differentially expressed in OSE_1_*-*OSE_0_ but not in control contrasts [*OSE*_1_*-expressed transcripts* (OSE_1_ex), Supplementary Table 5], 805 processes were significant after correction for multiple gene sets. Among them were the three previously highlighted GO terms (adjusted *p* < 2 × 10^−16^, Supplementary Table 4, Supplementary Figure 4). Furthermore, the gene sets *B cell mediated immunity* and *antigen processing and presentation* were significantly overrepresented not only in the analysis of CDT but also for the OSE_1_ex transcripts, indicating a potential role of B cells also in mildly affected OSE mice.

### Enrichment of MS Susceptibility Genes Among Transcripts Expressed in OSE

**Figure 2:**
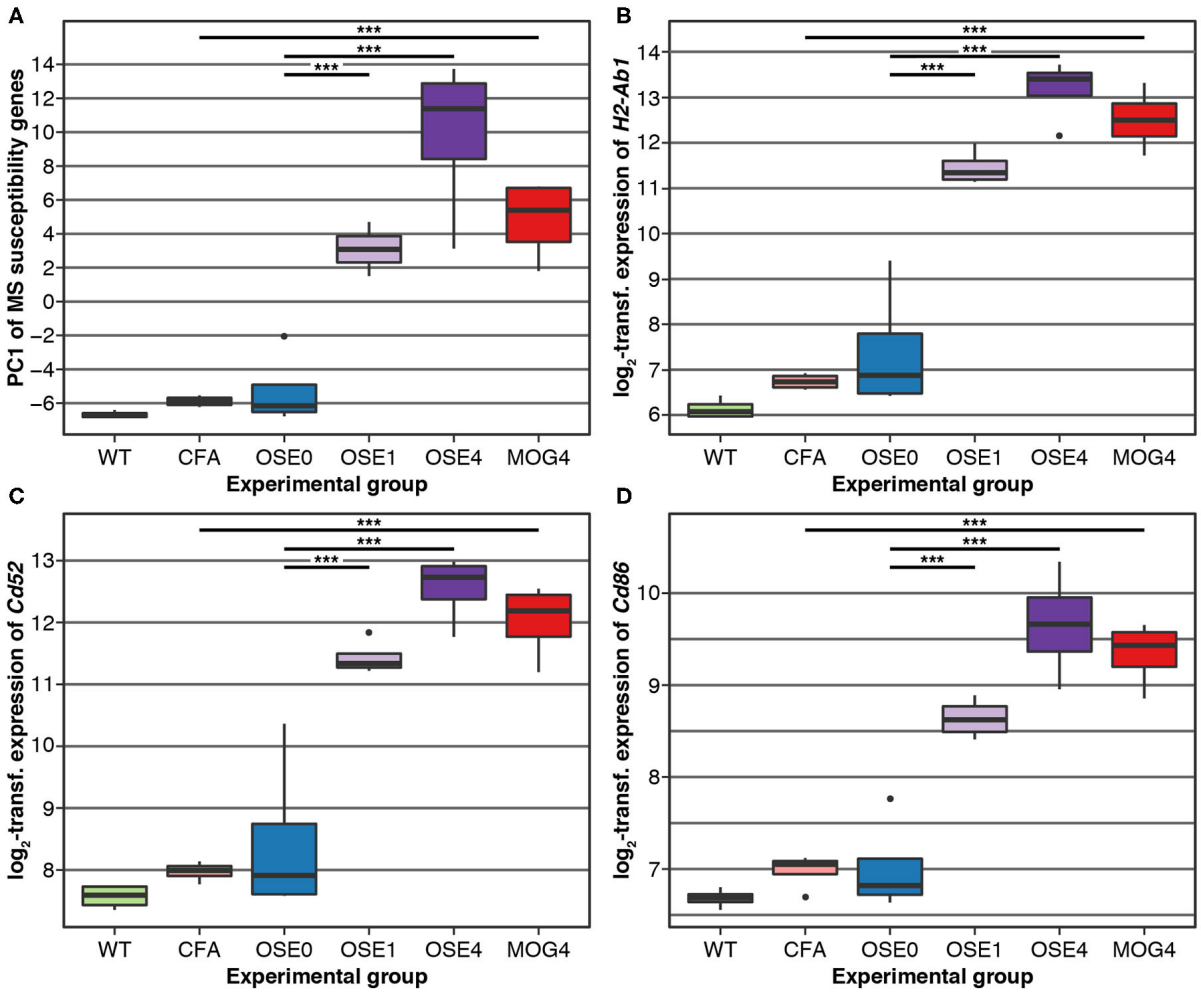
MS risk genes show a higher expression in diseased EAE animals. **(A)** Principal component analysis (PCA) of gene expression profiles of putative MS risk genes. Diseased mice showed higher MS risk gene expression levels (Supplementary Table 6). PC, principal component; y-axis unit, standard deviations. **(B–D)** Examples of expression levels of three putative MS risk genes, *H2-Ab1, Cd52*, and *Cd86* (Supplementary Table 2). In all three cases, diseased mice showed significantly higher expression levels, with the highest expression observed for OSE_4_ mice. Significance levels: *** adjusted *p* < 0.001.

Over 230 independent genetic loci associated with MS susceptibility in humans have been identified (1, 2). Based on these GWAS loci, 551 human MS susceptibility candidate genes have been proposed (2), for which expression data of 499 transcripts were available in our dataset. We conducted a PCA on these transcripts (265 genes) to analyze whether the expression of MS risk genes was increased in the EAE models. The first component, explaining 75.7% of the variance in expression of these transcripts, was significantly higher in all disease groups than in controls, indicating high expression levels of MS-associated genes in EAE, with the highest levels observed for OSE_4_ (Figure 2A, Supplementary Table 6). Also individual MS risk genes, e.g., *H2-Ab1, Cd52*, and *Cd86* (1, 2), as well as further putative MS-associated genes like *Cd74*, were among the transcripts showing the lowest differential expression *p*-values. They were significantly upregulated in all three diseased mouse types (Figures 1C,D, 2B–D, Supplementary Figure 5, Supplementary Table 2). Furthermore, differentially expressed genes from the analysis sets CDT, OSE_4_sp, and OSE_1_ex were significantly enriched for MS risk genes, while the MOG_4_sp genes were not (Table 1). OSE might thus be more closely connected to the etiology of human MS than MOG_35−55_ EAE is.

**Table 1:** Enrichment of MS susceptibility genes.

**DE transcript group**	**DE genes**	**Overlapping genes**	***p*-value**	**Adjusted *p*-value**
CDT	2,014	68	**<1** **×** **10**^**−5**^	**<4** **×** **10**^**−5**^
OSE_4_sp	2,362	68	**4.4** **×** **10**^**−4**^	**8.8** **×** **10**^**−4**^
MOG_4_sp	469	11	3.2 × 10^−1^	3.2 × 10^−1^
OSE_1_ex	693	34	**1.0** **×** **10**^**−5**^	**4.0** **×** **10**^**−5**^

*Of 551 genes considered, 265 were present in our data. P-values were computed using 100,000 permutations. Enrichments significant after Holm-Bonferroni correction for multiple testing (four tests) are highlighted in bold font (adjusted p-value < 0.05). DE, differentially expressed; WT, wildtype; CDT, common disease transcripts (differentially expressed for both contrasts OSE_4_-OSE_0_ and MOG_4_-CFA but not in the two control contrasts OSE_0_-WT or CFA-WT); OSE_4_sp, OSE_4_-specific transcripts (differentially expressed for the contrast OSE_4_-OSE_0_ but not in MOG_4_-CFA, OSE_0_-WT, or CFA-WT); MOG_4_sp, MOG_4_-specific transcripts (differentially expressed for the contrast MOG_4_-CFA but not in OSE_4_-OSE_0_, OSE_0_-WT, or CFA-WT); OSE_1_ex, OSE_1_-expressed transcripts (differentially expressed in OSE_1_-OSE_0_ but not in OSE_0_-WT or CFA-WT)*.

### Gene Expression in OSE Overlaps With T_H_ Cell-Specific Transcripts

**Figure 3:**
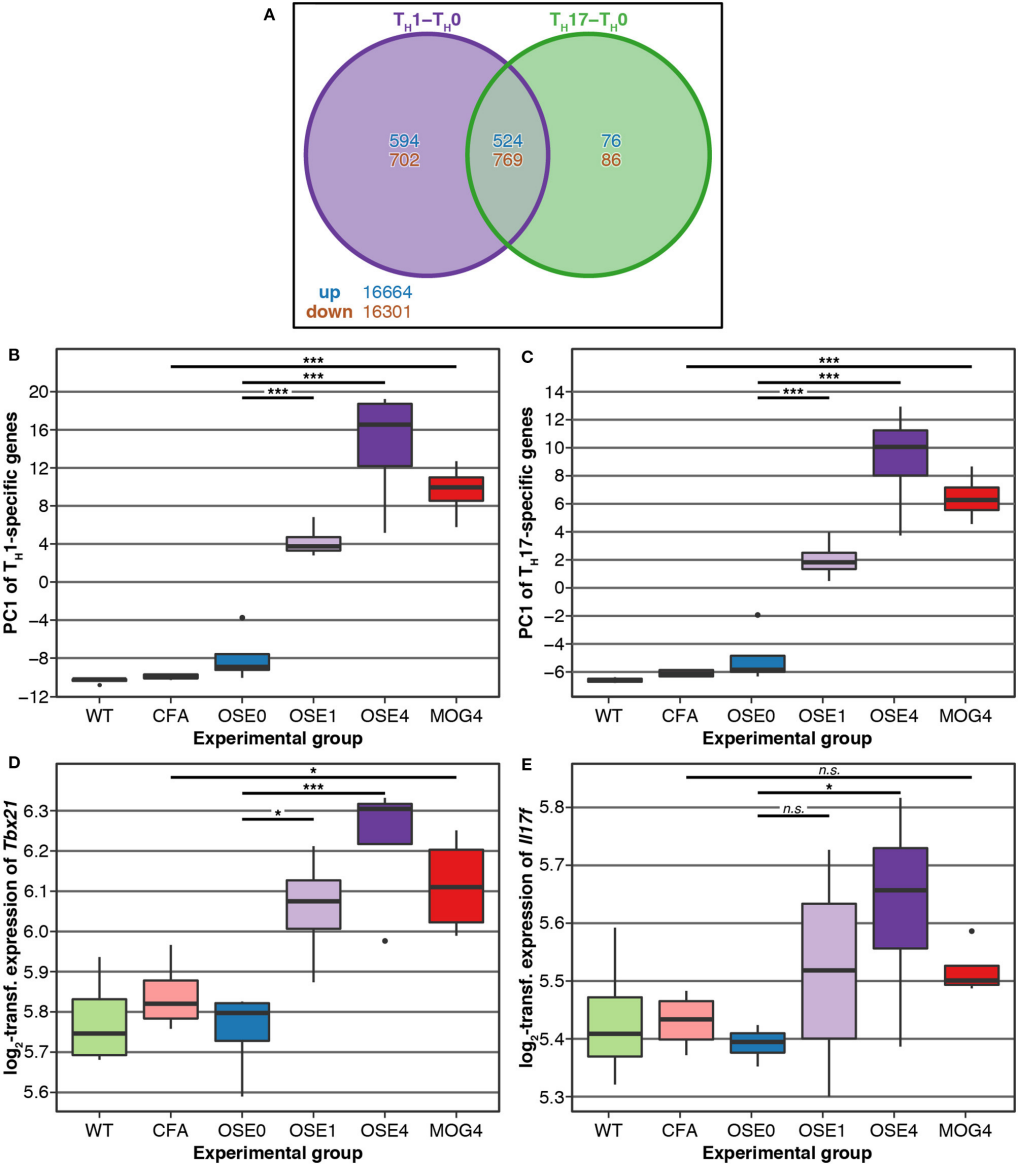
Genes differentially expressed in T_H_ cells show a higher expression in diseased EAE animals, especially in OSE_4_. **(A)** Venn diagram highlighting the number of transcripts differentially expressed in T_H_ cells. For this plot, up- and downregulated transcripts were analyzed separately, and transcripts differentially expressed in opposing directions are therefore included in the counts. **(B,C)** Principal component analysis (PCA) of gene expression profiles of transcripts differentially expressed in **(B)** T_H_1 and **(C)** T_H_17 cells. Diseased mice showed higher T_H_ cell-specific expression levels (Supplementary Table 7). PC, principal component; y-axis unit: standard deviations. **(D,E)** Examples of expression levels of the T_H_1 signature molecule **(D)** *Tbx21* and the T_H_17 signature molecule **(E)** *Il17f* . Diseased mice showed significantly higher expression levels of *Tbx21*, yet only OSE_4_ mice showed an increased expression of *Il17f* . Furthermore, the T_H_1 signature molecule *Ifng* was expressed significantly higher only in OSE_4_; the T_H_17 signature molecules *Rorc* and *Il17a* were not differentially expressed in any contrast (Supplementary Table 8). Significance levels: n.s.: *p* ≥ 0.05, * adjusted *p* < 0.05, *** adjusted *p* < 0.001.

T_H_ cell differentiation was identified as a key pathway in the etiology of MS (13). We, therefore, analyzed whether gene expression changes in EAE models were related to T_H_ cell differentiation. To this end, gene expression profiling of *in vitro* polarized T_H_1 and T_H_17 cells was conducted, derived from OSE mice. Compared to naïve T_H_0 cells, 8 × more transcripts were differentially expressed specifically in T_H_1 than in T_H_17 cells (Figure 3A). None of the transcripts differentially expressed in both T_H_1 and T_H_17 were regulated in opposite directions.

We examined via PCA whether the expression of T_H_1- and T_H_17-specific, differentially expressed probes was higher in EAE models than controls. The first component of T_H_1-and T_H_17-specific gene expression explained 49.6 and 68.6% of the variance, respectively. For both T_H_ cell types, the first component of cell-specific transcripts was significantly higher in all disease groups than in controls, with the highest levels for OSE_4_ (Figures 3B,C, Supplementary Table 7). Among signature molecules for T_H_1 cells, *Tbx21* (*T-bet*) was significantly upregulated in all diseased mice, *Ifng* only in OSE_4_ (Figure 3D, Supplementary Table 8). Of the examined T_H_17 markers, only *Il17f* was upregulated in OSE_4_, neither *Rorc* nor *Il17a* were differentially expressed (Figure 3E, Supplementary Table 8).

After correction for multiple testing, the CDT, OSE_4_sp, and OSE_1_ex analysis sets were significantly enriched for T_H_1- and T_H_17-specific transcripts (Table 2). In the case of MOG_4_sp transcripts, the overlap was lower and only significant for T_H_1-specific probes. These experiments indicate a stronger overlap of known MS-associated immune responses involving T_H_ cells with OSE than with MOG EAE.

**Table 2:** Enrichment of T_H_-specific transcripts.

**DE transcript group**	**DE genes**	**Cell type**	**Overlapping genes**	***p*-value**	**Adjusted *p*-value**
CDT	2,014	T_H_1	150	** <1** **×** **10**^**−5**^	** <8** **×** **10**^**−5**^
		T_H_17	28	**2.0** **×** **10**^**−2**^	**4.0** **×** **10**^**−2**^
OSE_4_sp	2,362	T_H_1	195	** <1** **×** **10**^**−5**^	** <8** **×** **10**^**−5**^
		T_H_17	36	**2.0** **×** **10**^**−3**^	**8.0** **×** **10**^**−3**^
MOG_4_sp	469	T_H_1	35	**1.1** **×** **10**^**−2**^	**3.3** **×** **10**^**−2**^
		T_H_17	7	9.8 × 10^−2^	9.8 × 10^−2^
OSE_1_ex	693	T_H_1	61	**2.0** **×** **10**^**−5**^	**1.2** **×** **10**^**−4**^
		T_H_17	16	**1.0** **×** **10**^**−3**^	**5.0** **×** **10**^**−3**^

*1,080 T_H_1- and 145 T_H_17-specific transcripts were examined. P-values were computed using 100,000 permutations. Enrichments significant after Holm-Bonferroni correction for multiple testing (eight tests) are highlighted in bold font (adjusted p-value < 0.05). DE, differentially expressed; WT, wildtype; CDT, common disease transcripts (differentially expressed for both contrasts OSE_4_-OSE_0_ and MOG_4_-CFA but not in the two control contrasts OSE_0_-WT or CFA-WT); OSE_4_sp, OSE_4_-specific transcripts (differentially expressed for the contrast OSE_4_-OSE_0_ but not in MOG_4_-CFA, OSE_0_-WT, or CFA-WT); MOG_4_sp, MOG_4_-specific transcripts (differentially expressed for the contrast MOG_4_-CFA but not in OSE_4_-OSE_0_, OSE_0_-WT, or CFA-WT); OSE_1_ex, OSE_1_-expressed transcripts (differentially expressed in OSE_1_-OSE_0_ but not in OSE_0_-WT or CFA-WT)*.

Finally, we analyzed whether EAE-associated genes differentially expressed in T_H_1 or T_H_17 cells were more closely connected to human MS. To this end, we intersected the lists of EAE-specific and T_H_-specific transcripts. Immune-related biological processes were overrepresented for CDT, OSE_4_sp, and OSE_1_ex genes intersected with T_H_1-specific genes. (Supplementary Table 9, Supplementary Figure 6). No terms were significantly overrepresented for any T_H_17-specific or MOG_4_sp genes.

CDT and OSE_1_ex genes differentially expressed in T_H_1 cells were significantly enriched for the IMSGC MS risk genes (*p* < 7 × 10^−4^, Table 3). The enrichment for OSE4sp did not withstand correction for multiple testing. Neither any of the T_H_17-specific gene sets nor the genes from the MOG_4_sp group were enriched for these risk genes. Thus, we conclude that OSE entails gene expression changes involving human MS gene risk genes, especially in T_H_1 cells, which were not observed to the same degree for MOG EAE.

**Table 3:** Enrichment of MS susceptibility genes among T_H_-specific transcripts.

**DE transcript group**	**Cell type**	**EAE T_**H**_ cell list size**	**Overlapping genes**	***p*-value**	**Adjusted *p*-value**
CDT	T_H_1	150	10	**6.5** **×** **10**^**−4**^	**5.2** **×** **10**^**−3**^
	T_H_17	30	3	2.1 × 10^−2^	1.1 × 10^−1^
OSE_4_sp	T_H_1	215	10	9.7 × 10^−3^	5.8 × 10^−2^
	T_H_17	41	3	4.7 × 10^−2^	1.6 × 10^−1^
MOG_4_sp	T_H_1	37	1	5.1 × 10^−1^	5.1 × 10^−1^
	T_H_17	7	1	1.3 × 10^−1^	2.6 × 10^−1^
OSE_1_ex	T_H_1	60	6	**1.1** **×** **10**^**−3**^	**7.7** **×** **10**^**−3**^
	T_H_17	16	2	3.9 × 10^−2^	1.6 × 10^−1^

*The p-values were computed using 100,000 permutations. Enrichments significant after Holm-Bonferroni correction for multiple testing (eight tests) are highlighted in bold font (adjusted p-value < 0.05). DE, differentially expressed; WT, wildtype; CDT, common disease transcripts (differentially expressed for both contrasts OSE_4_-OSE_0_ and MOG_4_-CFA but not in the two control contrasts OSE_0_-WT or CFA-WT); OSE_4_sp, OSE_4_-specific transcripts (differentially expressed for the contrast OSE_4_-OSE_0_ but not in MOG_4_-CFA, OSE_0_-WT, or CFA-WT); MOG_4_sp, MOG_4_ -specific transcripts (differentially expressed for the contrast MOG_4_-CFA but not in OSE_4_-OSE_0_, OSE_0_-WT, or CFA-WT); OSE_1_ex, OSE_1_-specific transcripts (differentially expressed in OSE_1_-OSE_0_ but not in OSE_0_-WT or CFA-WT)*.

## Discussion

With the identification of over 230 MS risk loci in recent GWAS, we move closer to understanding the etiology of MS. Further research relies on adequate animal models that have to be reassessed in the context of GWAS data. Given the interplay of genetics and environment in human MS, spontaneous EAE models like OSE might be more apt for studying the genetic risk component of MS than induced EAE models that require active experimental manipulation. In the present study, we performed spinal cord gene expression profiling to, first, characterize differences between spontaneous OSE and MOG-induced EAE and, second, to analyze the relationship of both models to human MS risk genes and T_H_ cell biology.

### OSE May Reflect the Etiology of MS Better Than MOG EAE Does

In comparison to MOG EAE, gene expression changes in OSE were stronger and more closely linked to immune pathways. This might reflect a more complex mode of disease induction in OSE than is the case for MOG EAE. OSE features active B and T cell cooperation, a mechanism highly relevant for the pathophysiology of human MS, as demonstrated by the effectivity of B cell-depleting treatments (10, 11). More than MOG EAE, OSE-specific transcripts were enriched for both human MS risk genes and T_H_ cell-specific transcripts and showed an overrepresentation of immune-specific gene sets. We thus hypothesize that OSE shows advantages over MOG EAE in studying the functional role of human MS risk genes and their associated immune pathways.

Nevertheless, many of the differentially expressed genes indicate that both EAE models faithfully recapitulate critical functional pathways of MS, especially regarding the role of antigen presentation and CD4^+^ T cells in MS immunopathogenesis (25, 26). Transcripts for the *HLA* genes *H2-Eb1* and *H2-Ab1*, homologous to *HLA-DRB5* and *HLA-DQB1*, were among the most differentially expressed probes. The alleles HLA-DRB5^*^01:01 and HLA-DQB1^*^06:02 are part of the *DR15-DQ6* haplotype and are, most likely because of linkage disequilibrium with HLA-DRB1^*^15:01, strongly associated with MS risk (27). In MS, memory B cells mediate autoproliferation of brain-homing T_H_1 cells in a HLA-DRB1^*^15:01-dependent manner (28). Interestingly, the antigen-presenting function of MOG-specific B cells is, in cooperation with T cells, important for the development of OSE (10). Among putative non-MHC MS risk genes (1, 2), *Cd86, Cd52*, and *Cd74* showed very robust support for differential expression.

We could thus show that EAE, and in particular OSE, constitutes a valuable model for studying the role of human MS risk genes. Several previous studies support this finding: First, humanized EAE models successfully replicated *HLA*-related risk variants, including HLA-DRB^*^15:01 (29). Second, knockout mice lacking the MS-associated *Il7r* are resistant to EAE (30). Third, shared human and EAE risk loci exist that are linked to T_H_ cell differentiation (31). Fourth, an overlap of upregulated genes between myelin-reactive T cells from MS patients and encephalitogenic CD4^+^ T cells isolated from EAE was described (32). Fifth, in a passive-transfer EAE study, several MS risk genes were suggested to be implicated in the transition from *in vitro*-generated MOG-specific T_H_17 cells to encephalitogenic CD4^+^ T cells (33).

Functional pathways involving MS risk genes interact with environmental factors to trigger an autoimmune response, as demonstrated by the role of epigenetic factors for MS risk (1, 34). Spontaneous EAE models might resemble gene-environment interactions more faithfully than MOG EAE does. For instance, in a spontaneous EAE model, disease onset could be prevented in mice kept under germ-free conditions (7). In this model, a higher incidence of EAE was observed following the transfer of the human gut microbiome from MS patients than when transferring the microbiome from the patient's healthy twin (8).

### T_H_1-Specific Transcripts Are Enriched for MS Risk Genes

Our gene set analyses point at a central role of lymphocyte activation in EAE induction and shed light on the ongoing controversy regarding the relative importance of T_H_1 and T_H_17 cells in mediating CNS autoimmunity (35). In accordance with previous studies (9), we observed a higher differential expression of selected T_H_1- than of T_H_17-specific transcripts in diseased mice. Interestingly, a high T_H_1/T_H_17 ratio is indicative of a lesion distribution pattern characterized by prominent spinal cord involvement, as is the case for both EAE models investigated in our study (12, 15, 36).

CDT and OSE_1_ex transcripts differentially expressed in T_H_1 cells were significantly enriched for MS risk genes (Table 3). We did not observe such an enrichment for transcripts differentially expressed in T_H_17 cells. Albeit also OSE_4_sp genes were only enriched for risk genes in T_H_1 cells at nominal significance (unadjusted *p* = 0.0097), T_H_1-expressed MOG_4_sp transcripts showed no trend for the enrichment of MS risk genes at all (unadjusted *p* = 0.51). In GO overrepresentation analyses, immune-related biological processes like *positive regulation of T cell proliferation* were significant for OSE_4_sp-genes differentially expressed in T_H_1 cells, but no GO gene sets at all were overrepresented in T_H_1-specific MOG_4_sp genes. In the context of T_H_1-driven immune responses, the OSE model might thus be linked more closely to human MS risk genes than MOG_35−55_ EAE is. However, T_H_17 cells can shift toward a T_H_1 phenotype in EAE (37, 38). The T_H_1 markers analyzed in the EAE models may, accordingly, reflect expression in a significant proportion of former T_H_17 cells. Therefore, our findings do not argue against a relevant impact of T_H_17 cells in either EAE model.

### Expression Patterns Across Different Disease Stages Can Be Studied Using OSE

Most genes differentially expressed in OSE_1_ mice were also recapitulated in severely affected OSE_4_ mice and showed the same direction of regulation in both disease stages. Many factors active in severe EAE thus also influence EAE during a mild or, potentially, early disease course. Effective immunotherapy is facilitated if the same biological pathways are continuously active throughout the entire disease. For example, the gene set *response to interferon-beta* was highly overrepresented in both OSE_1_ex and CDT and *Cd52* was differentially expressed in all diseased mice. Studying mild OSE cases might, therefore, constitute an interesting model for defining the initial triggers of MS and the identification of novel therapeutic options.

## Limitations

Our gene expression analysis of two EAE models had several limitations: First, the microarrays used covered only part of the murine transcriptome and thus, some MS risk genes could not be analyzed. Second, the statistical power of our analyses was restricted by the sample size. Third, the initial phases of EAE are hard to define since the disease develops over a short period. We thus analyzed mild OSE cases as a proxy for early disease. It is, however, unknown whether these animals would have developed more severe EAE later.

## Conclusions

Although hundreds of genetic MS risk loci have been identified, their functional role in the etiology of the disease still has to be resolved. Ideally, suitable animal models recapitulate molecular and functional pathways involving these genes. They may thus move research closer to the primary cause and etiology of MS, thereby supporting the identification of effective immunotherapies. No animal model fully reflects a heterogeneous human disease like MS and each EAE model available today only replicates a part of the human disease. Researchers will thus continue to study different aspects of MS using a variety of EAE models. Our results indicate that OSE, with its closer link to MS risk genes and T_H_ cell biology, may be better suited for studying the etiology of MS and for defining specific therapeutic targets than MOG-induced EAE is. Future studies will show whether OSE can fulfill this promise to model the human MS genetic risk landscape faithfully.

## Data Availability Statement

The datasets presented in this study can be found in online repositories. The names of the repository and accession number(s) can be found at: https://www.ebi.ac.uk/arrayexpress/, E-MTAB-9132; https://www.ebi.ac.uk/arrayexpress/, E-MTAB-9133.

## Ethics Statement

The animal study was reviewed and approved by Tierschutzkommission der Regierung von Oberbayern, Munich, Germany.

## Author Contributions

HF, GK, PW, and FW contributed to the original conception and design of the study. HF, GK, and PW conducted experiments. DK and TA devised the statistical analyses. DK, BP, and TA conducted statistical analyses. BM-M and FW supervised the study. HF and TA drafted the manuscript. All authors contributed to manuscript revision, read, and approved the submitted version.

## Conflict of Interest

The authors declare that the research was conducted in the absence of any commercial or financial relationships that could be construed as a potential conflict of interest.

## Supplementary Material

Supplementary Figure 1 Disease course of MOG EAE in *C57BL/6* mice. MOG EAE was induced in *C57BL/6* mice as described in the Methods. The plot shows mean clinical scores (*n* = 4) and the standard error of the mean. The disease score of MOG EAE mice began to increase on day ten. The mice were sacrificed when they reached a score of 4. Control mice (CFA) consistently remained at a disease score of 0.

Supplementary Figure 2 Efficiency of T_H_1 and T_H_17 differentiation. **(A)** T cells from OSE mice were activated under T_H_1- and T_H_17-polarizing conditions and analyzed for intracellular IL-17 and IFN-γ cytokine expression by flow cytometry. The data represent the percentage of cytokine-producing cells in the gated CD4^+^ populations of naïve (T_H_0), T_H_1, and T_H_17 cells. **(B)** Quantification of IL-17 and IFN-γ in the culture supernatants of T_H_1 and T_H_17 cells by ELISA. The plots show the mean and the standard error of the mean (SEM). **(C)** IL-17 and IFN-γ mRNA expression of naïve (T_H_0), T_H_1-, and T_H_17-polarized cells quantified by real-time PCR. The data are representative of three independent experiments. The plots show the mean and SEM.

Supplementary Figure 3 Overrepresented immune system pathways in OSE and MOG EAE contrasts. The plots show overrepresented GO terms that are descendants of the term *Immune System Process* (Supplementary Table 4) for the contrasts **(A)** OSE_1_*-*OSE_0_, **(B)** OSE_4_*-*OSE_0_, **(C)** MOG_4_*-*CFA, and **(D)** MOG_4_*-*OSE_4_. The -log_10_(FDR) from hypergeometric tests is shown on the x-axis and used for coloring the plots (darker colors represent lower FDRs).

Supplementary Figure 4 The top 40 overrepresented immune system pathways in the differentially expressed transcripts groups. The plots show the top 40 overrepresented GO terms that are descendants of the term *Immune System Process* (Supplementary Table 4) for the transcript groups **(A)** CDT, common disease transcripts (differentially expressed for both contrasts OSE_4_*-*OSE_0_ and MOG_4_*-*CFA but not in the two control contrasts OSE_0_-WT or CFA-WT), **(B)** OSE_4_sp, OSE_4_-specific transcripts (differentially expressed for the contrast OSE_4_*-*OSE_0_ but not in MOG_4_*-*CFA, OSE_0_-WT, or CFA-WT), **(C)** OSE_1_ex, OSE_1_-expressed transcripts (differentially expressed in OSE_1_*-*OSE_0_ but not in OSE_0_-WT or CFA-WT). Note that no GO terms that are descendants of the term *Immune System Process* were significantly overrepresented for the group MOG_4_sp, MOG_4_-specific transcripts (differentially expressed for the contrast MOG_4_*-*CFA but not in OSE_4_*-*OSE_0_, OSE_0_-WT, or CFA-WT). The -log_10_(FDR) from hypergeometric tests is shown on the x-axis and used for coloring the plots (darker colors represent lower FDRs).

Supplementary Figure 5 Expression levels of *Cd74* and *Icam1*. Diseased mice showed an increased expression of **(A)** *Cd74* and **(B)** *Icam1* (Supplementary Table 2). Significance levels: ^*^ adjusted *p* < 0.05, ^**^ adjusted *p* < 0.01, ^***^ adjusted *p* < 0.001.

Supplementary Figure 6 The top 40 overrepresented immune system pathways in the differentially expressed transcripts groups intersected with T_H_1-specific genes. The plots show the top 40 overrepresented GO terms that are descendants of the term *Immune System Process* (Supplementary Table 9) for the transcript groups **(A)** CDT (common disease transcripts) intersected with T_H_1-specific genes, **(B)** OSE_4_sp (OSE_4_-specific transcripts) intersected with T_H_1-specific genes, **(C)** OSE_1_ex (OSE_1_-expressed transcripts) intersected with T_H_1-specific genes. Note that no GO terms were significantly overrepresented for any T_H_17-specific or MOG_4_sp genes. The -log_10_(FDR) from hypergeometric tests is shown on the x-axis and used for coloring the plots (darker colors represent lower FDRs).

Supplementary Table 1 Results from k-means clustering (Figure 1A).

Supplementary Table 2 Differential expression results for all analyzed contrasts. FC, fold change; CI, 95% confidence interval.

Supplementary Table 3 Comparison of fold changes in expression levels between contrasts (Figures 1C,D). CI, 95% confidence interval.

Supplementary Table 4 Results from WebGestalt gene ontology (GO) over-representation analyses (ORA) for the analysis groups CDT, OSE_4_sp, MOG_4_sp, and OSE_1_ex (see the legend of Table 1 and Supplementary Figure 5) as well as the contrasts OSE_1_*-*OSE_0_, OSE_4_*-*OSE_0_, MOG_4_*-*CFA, and MOG_4_*-*OSE_4_ (Supplementary Figure 4). FDR, 5% false discovery rate. Gene sets that are labeled in bold if they are descendants of the GO term “Immune System Process”.

Supplementary Table 5 Differential expression results for the analysis groups (see the legend of Table 1) CDT, CDT, OSE_4_sp, MOG_4_sp, OSE_1_ex, and OSE_1_sp. OSE_1_sp transcripts were differentially expressed in OSE_1_*-*OSE_0_ but not in any other contrast. FC, fold change, CI, 95% confidence interval. For CDT transcripts, separate coefficients are provided for the two contrasts MOG_4_-CFA and OSE_4_-OSE_0_.

Supplementary Table 6 Results from the principal component analysis (PCA) of gene expression profiles of putative MS risk genes (Figure 2A). FC, fold change, CI, 95% confidence interval. *P*-values were adjusted for multiple testing using Holm-Bonferroni correction.

Supplementary Table 7 Results from the principal component analysis (PCA) of gene expression profiles of T_H_ cell-specific transcripts (Figures 3B,C). FC, fold change; CI, 95% confidence interval. *P*-values were adjusted for multiple testing using Holm-Bonferroni correction.

Supplementary Table 8 Differential expression results for selected T_H_1 and T_H_17 signature molecules (Figures 3D,E). DE, differentially expressed.

Supplementary Table 9 Results from Webgestalt gene ontology (GO) over-representation analyses (ORA) for the analysis groups CDT, OSE_4_sp, and OSE_1_ex (see the legend of Table 1) among T_H_1-specific transcripts (Supplementary Figure 6). For MOG_4_sp or T_H_17-specific transcripts, no significant gene sets were found. FDR, 5% false discovery rate. Gene sets that are labeled in bold if they are descendants of the GO term “Immune System Process”.
