# Supplementary material for: Gene Expression in Spontaneous Experimental Autoimmune Encephalomyelitis Is Linked to Human Multiple Sclerosis Risk Genes
Source: Front Immunol. 2020 Sep 18;11:2165. doi: 10.3389/fimmu.2020.02165 (PMC7531036; doi:10.3389/fimmu.2020.02165)
Supplement: Supplementary file 1 [file Data_Sheet_1.PDF]

Supplementary Material

1 Supplementary Methods

ELISA

Culture supernatants were collected from polarized cells and frozen at -20 ºC until quantification. Cytokine levels were measured using matching antibody pairs for IFN-γ (purified capture antibody clone AN-18, biotinylated detection antibody clone XMG1.2, BD Pharmingen) and IL-17 (purified capture antibody clone eBio17CK15A5, biotinylated detection antibody clone eBio17B7, eBioscience).

Quantitative real-time PCR

Total RNA was isolated with the TRI Reagent extraction method and reverse-transcribed into cDNA using oligo-dT primers and SuperScript II Reverse Transcriptase (Invitrogen) according to the manufacturer's instructions. Primers and probe sequences are shown below. For amplification, the ABsolute QPCR mix was used (ABgene). Each reaction was run in triplicates on an ABI 7900 machine (Applied Biosystems) and was normalized to transcripts of the housekeeping gene *GAPDH*. The primary data was analyzed with the GeneAmp SDS v2.3 software (Applied Biosystems).

Gene	Primer name	Oligo sequence (5' to 3')
IFN-γ	IFN-γ sense	TCAAGTGGCATAGATGTGGAAGAA
	IFN-γ anti-sense	TGGCTCTGCAGGATTTTCATG
	IFN-γ probe	FAM-TCACCATCCTTTTGCCAGTTCCTCCAG-TAMRA
IL-17A	IL-17 sense	AACTCCCTTGGCGCAAAAGT
	IL-17 anti-sense	GGCACTGAGCTTCCCAGATC
	IL-17 probe	FAM-CCACGTCACCCTGGACTCTCCACC-TAMRA
GAPDH	GAPDH sense	TCACCACCATGGAGAAGGC
	GAPDH anti-sense	GCTAAGCAGTTGGTGGTGCA
	GAPDH probe	FAM-ATGCCCCCATGTTTGTGATGGGTGT-TAMRA
