# Supplementary figures and images for: Gene Expression in Spontaneous Experimental Autoimmune Encephalomyelitis Is Linked to Human Multiple Sclerosis Risk Genes
Source: Front Immunol. 2020 Sep 18;11:2165. doi: 10.3389/fimmu.2020.02165 (PMC7531036; doi:10.3389/fimmu.2020.02165)

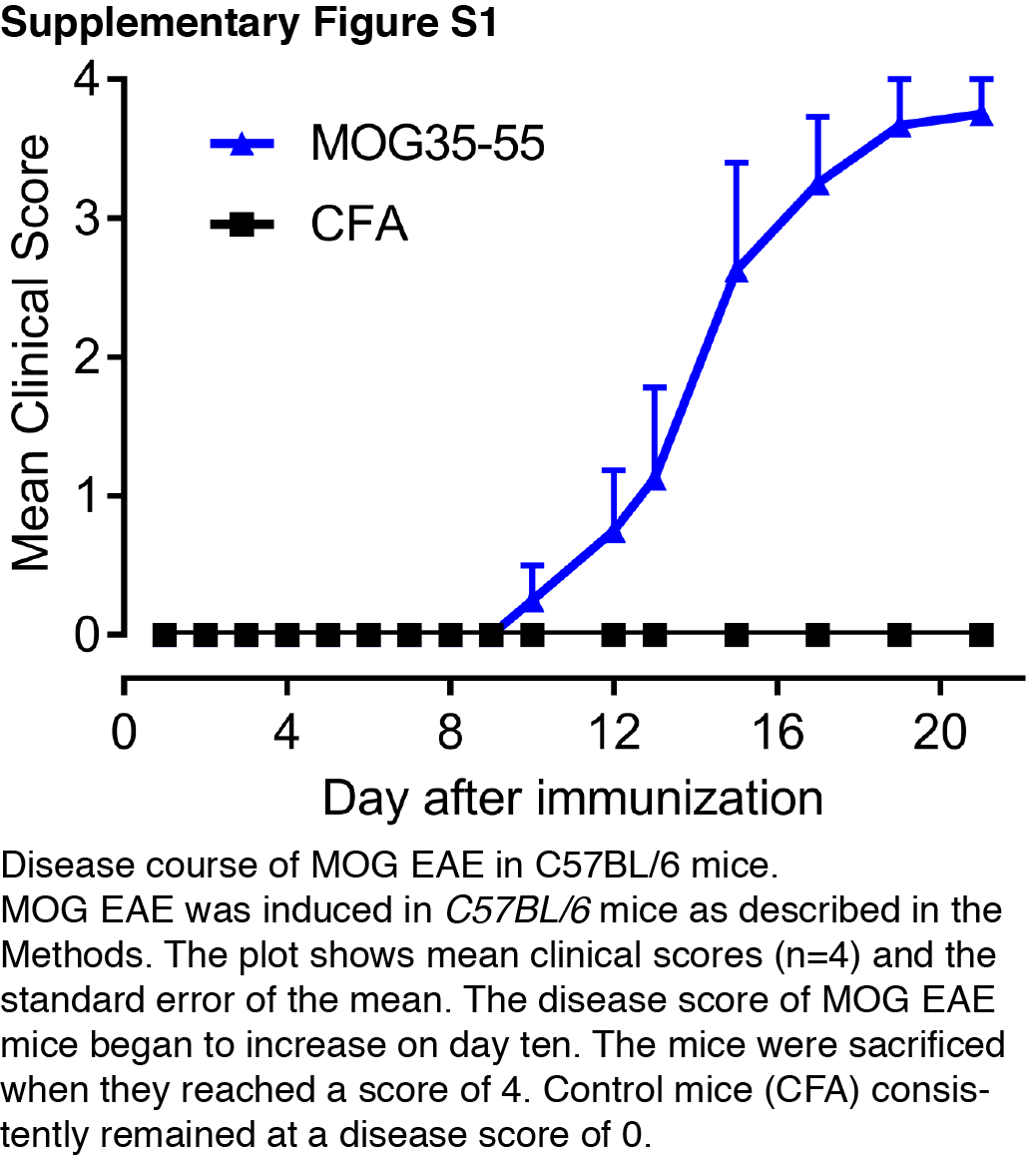

Supplement: Supplementary Figure 1 — Disease course of MOG EAE in C57BL/6 mice. MOG EAE was induced in C57BL/6 mice as described in the Methods. The plot shows mean clinical scores (n = 4) and the standard error of the mean. The disease score of MOG EAE mice began to increase on day ten. The mice were sacrificed when they reached a score of 4. Control mice (CFA) consistently remained at a disease score of 0. [file Image_1.PNG]

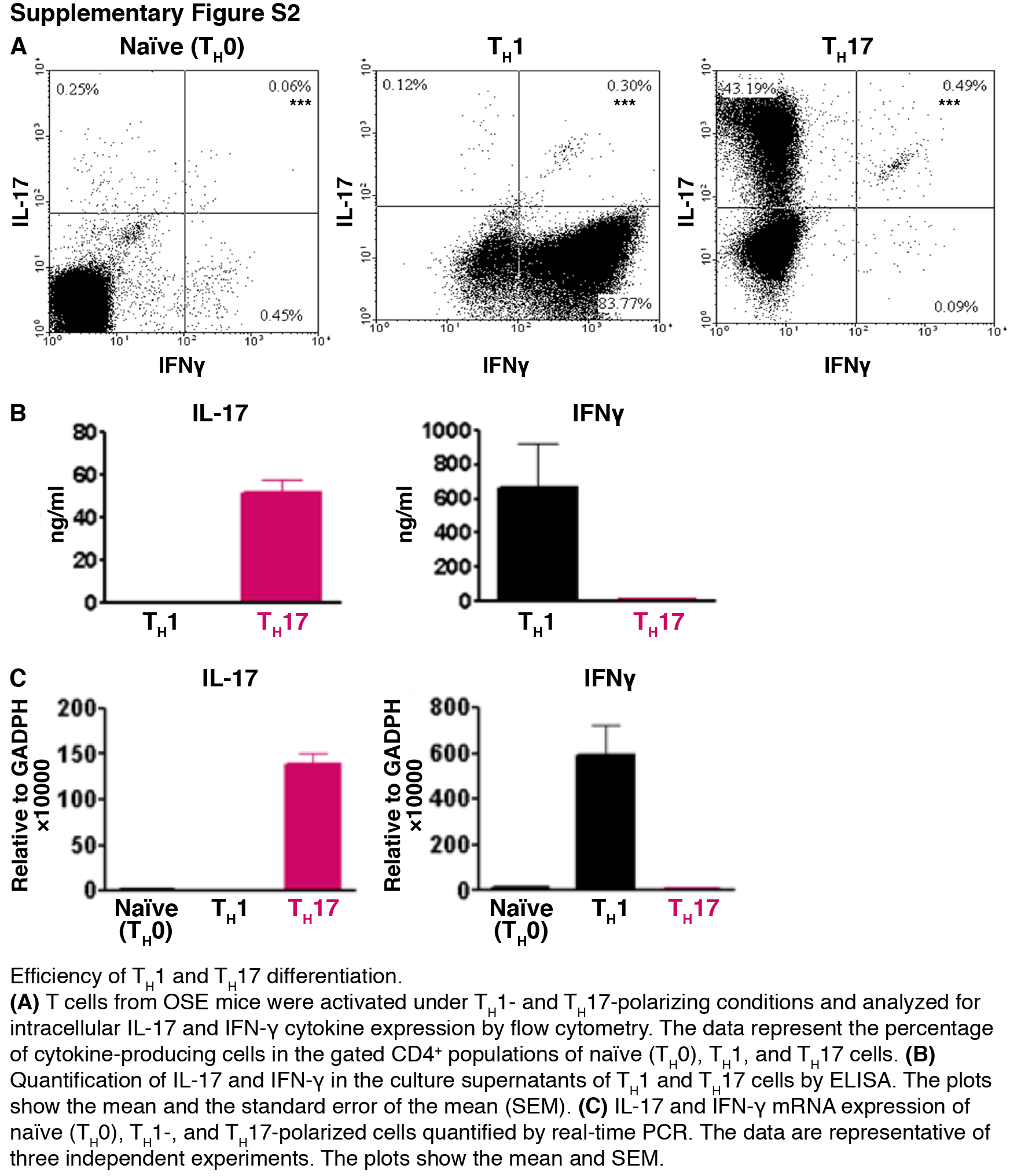

Supplement: Supplementary Figure 2 — Efficiency of TH1 and TH17 differentiation. (A) T cells from OSE mice were activated under TH1- and TH17-polarizing conditions and analyzed for intracellular IL-17 and IFN-γ cytokine expression by flow cytometry. The data represent the percentage of cytokine-producing cells in the gated CD4+ populations of naïve (TH0), TH1, and TH17 cells. (B) Quantification of IL-17 and IFN-γ in the culture supernatants of TH1 and TH17 cells by ELISA. The plots show the mean and the standard error of the mean (SEM). (C) IL-17 and IFN-γ mRNA expression of naïve (TH0), TH1-, and TH17-polarized cells quantified by real-time PCR. The data are representative of three independent experiments. The plots show the mean and SEM. [file Image_2.PNG]

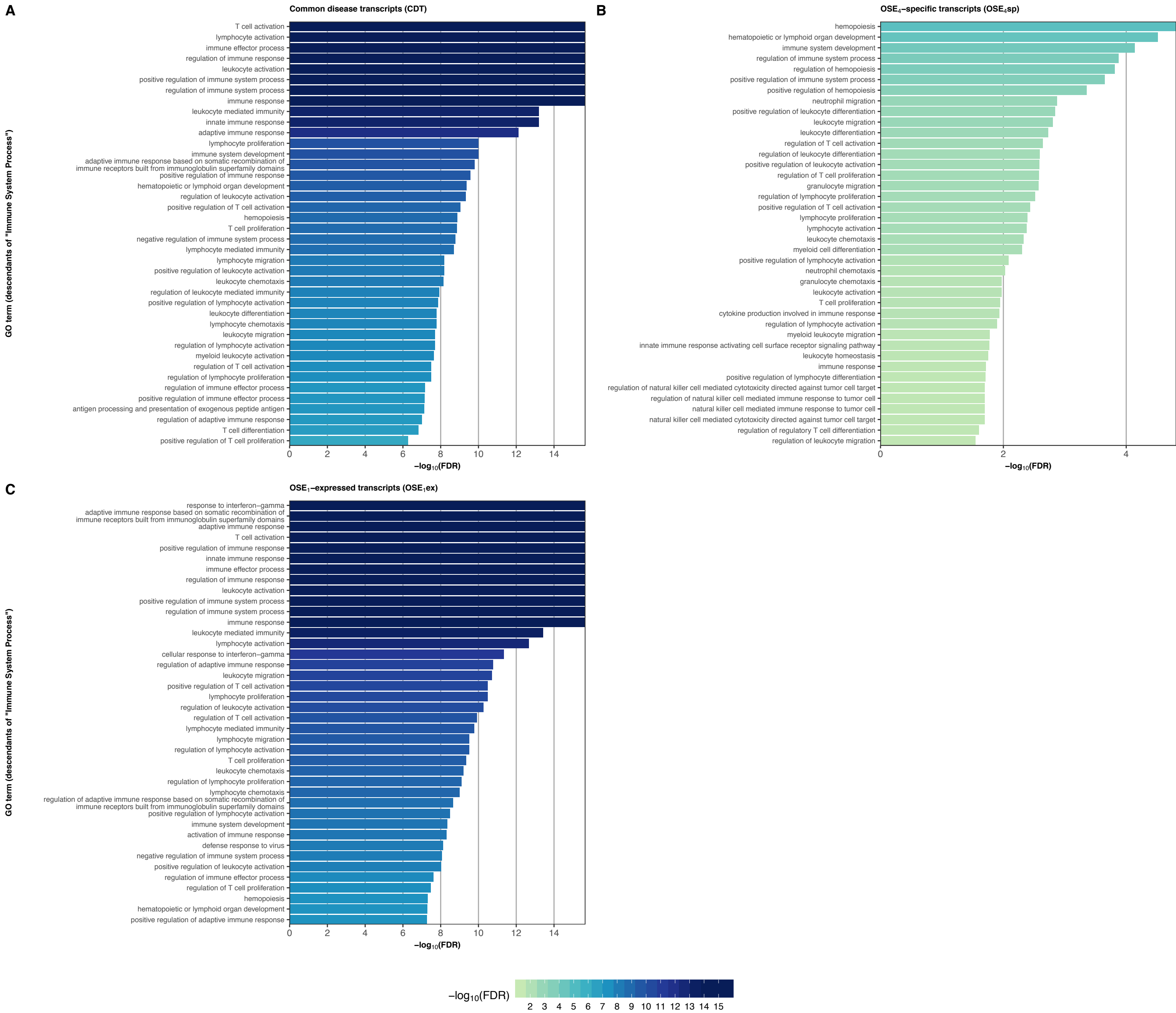

Supplement: Supplementary Figure 4 — The top 40 overrepresented immune system pathways in the differentially expressed transcripts groups. The plots show the top 40 overrepresented GO terms that are descendants of the term Immune System Process (Supplementary Table 4) for the transcript groups (A) CDT, common disease transcripts (differentially expressed for both contrasts OSE4-OSE0 and MOG4-CFA but not in the two control contrasts OSE0-WT or CFA-WT), (B) OSE4sp, OSE4-specific transcripts (differentially expressed for the contrast OSE4-OSE0 but not in MOG4-CFA, OSE0-WT, or CFA-WT), (C) OSE1ex, OSE1-expressed transcripts (differentially expressed in OSE1-OSE0 but not in OSE0-WT or CFA-WT). Note that no GO terms that are descendants of the term Immune System Process were significantly overrepresented for the group MOG4sp, MOG4-specific transcripts (differentially expressed for the contrast MOG4-CFA but not in OSE4-OSE0, OSE0-WT, or CFA-WT). The -log10(FDR) from hypergeometric tests is shown on the x-axis and used for coloring the plots (darker colors represent lower FDRs). [file Image_4.PDF]
