# Supplementary material for: Gene Expression in Spontaneous Experimental Autoimmune Encephalomyelitis Is Linked to Human Multiple Sclerosis Risk Genes
Source: Front Immunol. 2020 Sep 18;11:2165. doi: 10.3389/fimmu.2020.02165 (PMC7531036; doi:10.3389/fimmu.2020.02165)
Supplement: Supplementary Figure 3 — Overrepresented immune system pathways in OSE and MOG EAE contrasts. The plots show overrepresented GO terms that are descendants of the term Immune System Process (Supplementary Table 4) for the contrasts (A) OSE1-OSE0, (B) OSE4-OSE0, (C) MOG4-CFA, and (D) MOG4-OSE4. The -log10(FDR) from hypergeometric tests is shown on the x-axis and used for coloring the plots (darker colors represent lower FDRs). [file Image_3.PDF]

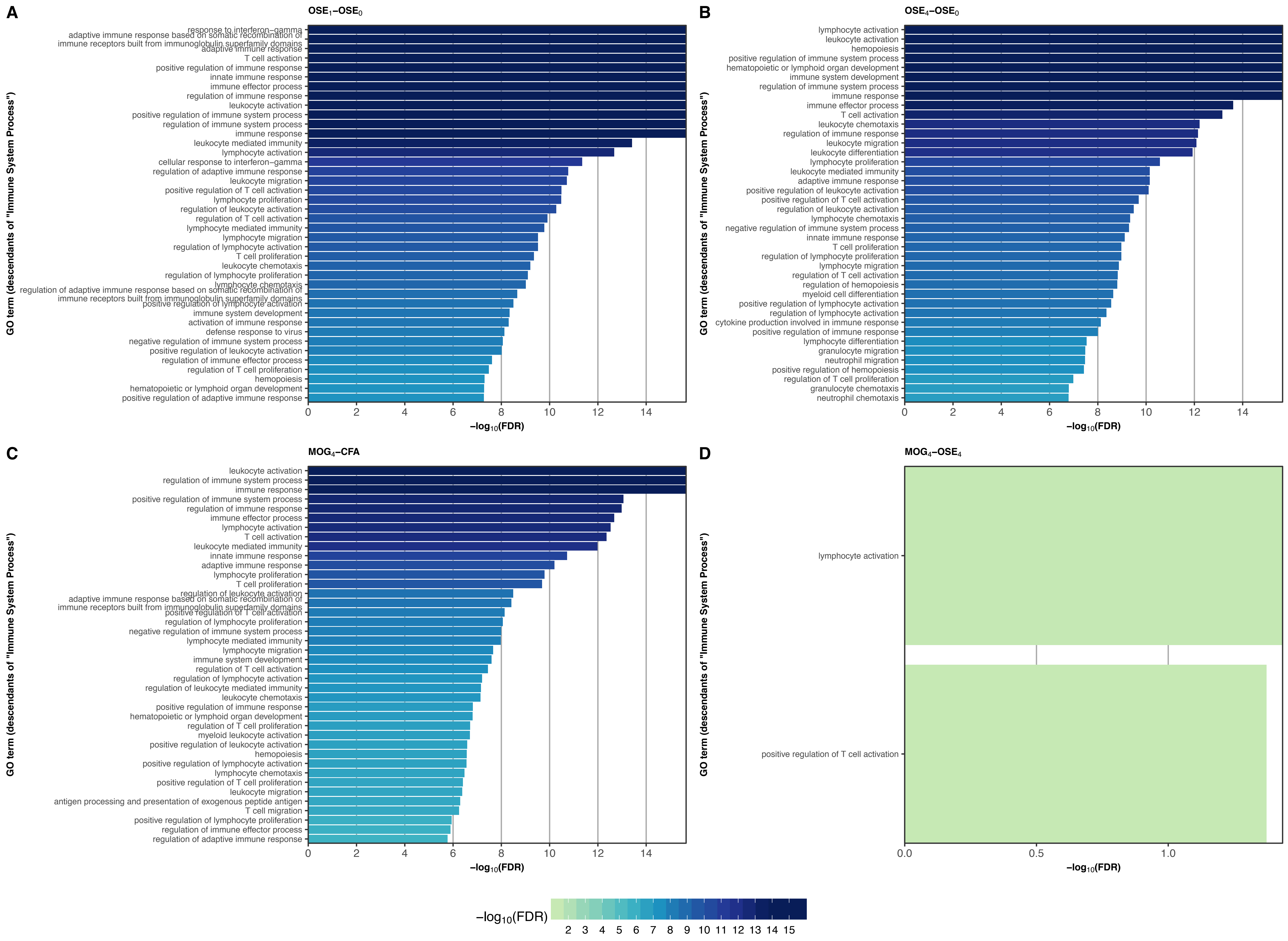

Supplementary Figure S3: Overrepresented immune system pathways in OSE and MOG EAE contrasts.The plots show overrepresented GO terms that are descendants of the term *Immune System Process* (Supplementary Table S4) for the contrasts **(A)** OSE1-OSE0, **(B)** OSE4-OSE0, **(C)** MOG4-CFA, and **(D)** MOG4-OSE4. The -log10(FDR) from hypergeometric tests is shown on the x-axis and used for coloring the plots (darker colors represent lower FDRs).
