# Supplementary material for: Gene Expression in Spontaneous Experimental Autoimmune Encephalomyelitis Is Linked to Human Multiple Sclerosis Risk Genes
Source: Front Immunol. 2020 Sep 18;11:2165. doi: 10.3389/fimmu.2020.02165 (PMC7531036; doi:10.3389/fimmu.2020.02165)
Supplement: Supplementary Figure 5 — Expression levels of Cd74 and Icam1. Diseased mice showed an increased expression of (A) Cd74 and (B) Icam1 (Supplementary Table 2). Significance levels: * adjusted p < 0.05, ** adjusted p < 0.01, *** adjusted p < 0.001. [file Image_5.PDF]

Supplementary Figure S5: Expression levels of *Cd74* and *Icam1*

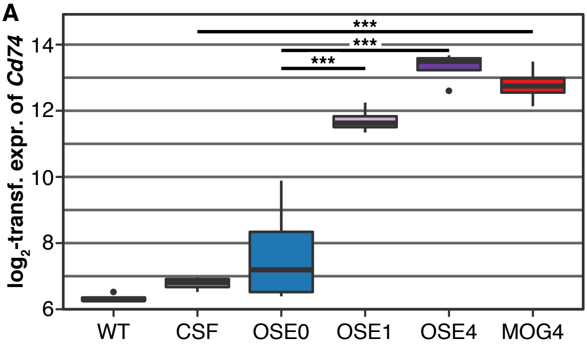

Diseased mice showed an increased expression of *Cd74* (Supplementary Table S2).
Significance levels: * adjusted $p<0.05$, ** adjusted $p<0.01$, *** adjusted $p<0.001$.

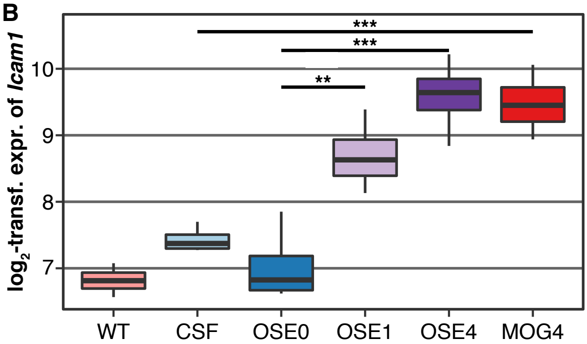

Diseased mice showed an increased expression of *Icam1* (Supplementary Table S2).
Significance levels: * adjusted $p<0.05$, ** adjusted $p<0.01$, *** adjusted $p<0.001$.
