# Supplementary material for: Gene Expression in Spontaneous Experimental Autoimmune Encephalomyelitis Is Linked to Human Multiple Sclerosis Risk Genes
Source: Front Immunol. 2020 Sep 18;11:2165. doi: 10.3389/fimmu.2020.02165 (PMC7531036; doi:10.3389/fimmu.2020.02165)
Supplement: Supplementary Figure 6 — The top 40 overrepresented immune system pathways in the differentially expressed transcripts groups intersected with TH1-specific genes. The plots show the top 40 overrepresented GO terms that are descendants of the term Immune System Process (Supplementary Table 9) for the transcript groups (A) CDT (common disease transcripts) intersected with TH1-specific genes, (B) OSE4sp (OSE4-specific transcripts) intersected with TH1-specific genes, (C) OSE1ex (OSE1-expressed transcripts) intersected with TH1-specific genes. Note that no GO terms were significantly overrepresented for any TH17-specific or MOG4sp genes. The -log10(FDR) from hypergeometric tests is shown on the x-axis and used for coloring the plots (darker colors represent lower FDRs). [file Image_6.PDF]

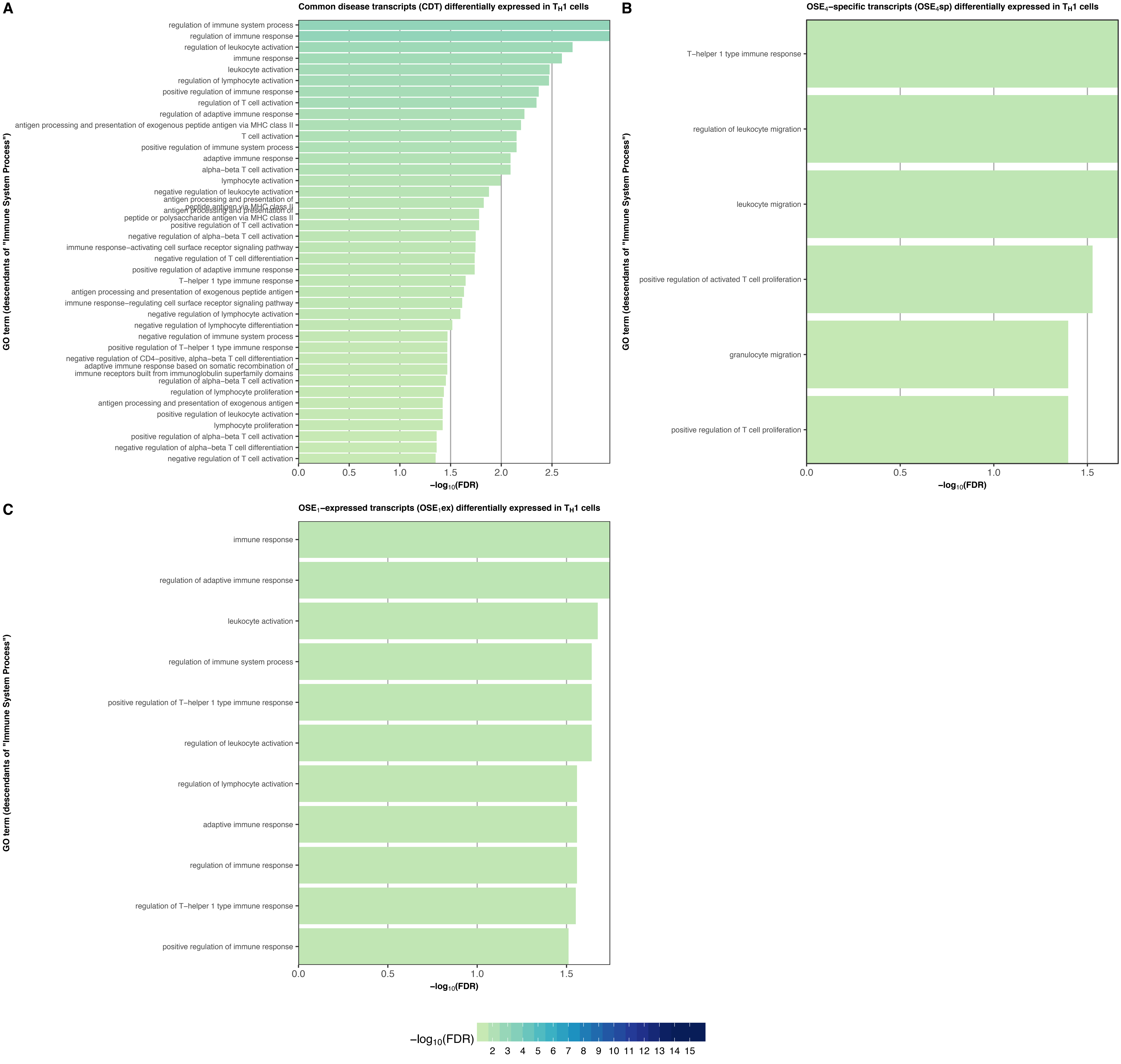

Supplementary Figure S6: The top 40 overrepresented immune system pathways in the differentially expressed transcripts groups intersected with T_H1-specific genes.
The plots show the top 40 overrepresented GO terms that are descendants of the term *Immune System Process* (Supplementary Table S9) for the transcript groups **(A)** CDT (common disease transcripts) intersected with T_H1-specific genes, **(B)** OSE4sp (OSE4-specific transcripts) intersected with T_H1-specific genes, **(C)** OSE1ex (OSE1-expressed transcripts) intersected with T_H1-specific genes.
Note that no GO terms were significantly overrepresented for any T_H17-specific or MOG4sp genes.
The -log10(FDR) from hypergeometric tests is shown on the x-axis and used for coloring the plots (darker colors represent lower FDRs).
